# Natural products from *Zanthoxylum heitzii* with potent activity against the malaria parasite

**DOI:** 10.1186/s12936-016-1533-x

**Published:** 2016-09-20

**Authors:** Christopher Dean Goodman, Ingvild Austarheim, Vanessa Mollard, Bertin Mikolo, Karl Egil Malterud, Geoffrey I. McFadden, Helle Wangensteen

**Affiliations:** 1 School of BioSciences, University of Melbourne, Parkville, VIC 3010 Australia; 2 Department of Pharmaceutical Chemistry, School of Pharmacy, University of Oslo, P. O. Box 1068, Blindern, 0316 Oslo, Norway; 3 National Polytechnic High School, Marien Ngouabi University, BP 69 Brazzaville, Republic of Congo

**Keywords:** *Zanthoxylum heitzii*, Rutaceae, Dihydronitidine, Anti-malarial, Benzophenanthridine alkaloid, Ookinete, Transmission blocking, Malaria, Ethnobotany

## Abstract

**Background:**

*Zanthoxylum heitzii* (Rutaceae) (olon) is used in traditional medicine in Central and West Africa to treat malaria. To identify novel compounds with anti-parasitic activity and validate medicinal usage, extracts and compounds isolated from this tree were tested against the erythrocytic stages of the human malaria parasite *Plasmodium falciparum* and for inhibition of transmission in rodent malaria parasite *Plasmodium berghei.*

**Results:**

Hexane bark extract showed activity against *P. falciparum* (IC_50_ 0.050 μg/ml), while leaf and seed extracts were inactive. Fractionation of the hexane bark extract led to the identification of three active constituents; dihydronitidine, pellitories and heitziquinone. Dihydronitidine was the most active compound with an IC_50_ value of 0.0089 µg/ml (25 nM). This compound was slow acting, requiring 50 % longer exposure time than standard anti-malarials to reach full efficacy. Heitziquinone and pellitorine were less potent, with IC_50_ values of 3.55 μg/ml and 1.96 µg/ml, but were fast-acting. *Plasmodium berghei* ookinete conversion was also inhibited by the hexane extract (IC_50_ 1.75 µg/ml), dihydronitidine (0.59 µg/ml) and heitziquinone (6.2 µg/ml). Water extracts of *Z. heitzii* bark contain only low levels of dihydronitidine and show modest anti-parasitic activity.

**Conclusions:**

Three compounds with anti-parasitic activity were identified in *Z. heitzii* bark extract. The alkaloid dihydronitidine is the most effective of these, accounting for the bulk of activity in both erythrocytic and transmission-blocking assays. These compounds may present good leads for development of novel anti-malarials and add to the understanding of the chemical basis of the anti-parasitic activity in these classes of natural product.

**Electronic supplementary material:**

The online version of this article (doi:10.1186/s12936-016-1533-x) contains supplementary material, which is available to authorized users.

## Background

In many African countries with high rates of malaria transmission, there is a common tradition of using native plants to treat malaria. The most seriously affected countries are associated with a dysfunctional health system and poverty [1], and accessibility to anti-malarial treatment is often inadequate or non-existent. Where available, free artemisinin combination therapy (ACT) is limited to pregnant women and children under 5 years of age [1], making expenditure on malaria treatment a huge burden for middle-income households and essentially unaffordable for most of the population who live on less than $2 a day [2]. Substandard and counterfeit anti-malarials are also major concerns and contribute to drug resistance [3, 4]. Use of herbal anti-malarials in sub-Saharan Africa is widespread, although the reported prevalence varies considerably. A meta-analysis from 2004 indicates an overall prevalence of 24 %, but regional use varied from 0 to 75 % [5]. Use of traditional medicine is most prevalent in rural and poor areas where it plays a very important role for disease control [6]. Traditional medicines have provided the basis for highly effective anti-malarials: the quinine and artemisinin derivatives. With resistance to artemisinin derivatives currently on the rise [7], identifying novel anti-malarials and understanding the efficacy of traditional remedies is of increasing importance.

Members of the genus *Zanthoxylum* are widely used as traditional medicines for the treatment and prevention of malaria [8–10]. *Zanthoxylum* spp. are well known for producing an array of complex secondary metabolites, especially benzophenanthridine alkaloids, some of which have previously been shown to have anti-malarial activity [11–14]. The in vitro efficacy of these compounds lends support for their use in traditional medicine. The tree *Zanthoxylum heitzii* (syn*. Fagara heitzii*), grows over a range that encompasses many areas of high malaria transmission in Central-West Africa [10]. In this area, the bark is traditionally used to treat malaria [10]. *Zanthoxylum heitzii* bark extracts and major components of the extracts have previously been shown to have insecticidal activity [16–18] but it is unknown if *Z. heitzii* contains any compounds with significant anti-parasitic activity.

This paper investigates the activity of extracts and chemical components isolated from *Z. heitzii* against the malaria parasites, *Plasmodium falciparum* and *Plasmodium berghei* to investigate the scientific rationale behind the traditional use against malaria. Fractionation and in vitro testing against *P. falciparum* identified three compounds with significant anti-parasitic activity, with one, dihydronitidine, being active at low nanomolar concentrations.

## Methods

### Plant material

Stem bark, seeds and leaves of *Zanthoxylum heitzii* (Rutaceae) were harvested in Douakani, Republic of Congo during November 2011. Voucher samples are kept in the Section of Pharmacognosy, School of Pharmacy, University of Oslo, registry number ZH-B-111202 (bark), ZH-L-111201 (leaf) and ZH-S-111203 (seed). This work was carried out in accordance with the Nagoya convention.

### Extraction and isolation of natural compounds

The plant material was extracted and natural products isolated according to Moussavi et al. [18] and Wangensteen et al. [19]. Briefly, the stem bark, seeds and leaves were air dried and made into a powder (<1 mm). The powdered plant materials were first extracted with hexane in a Soxhlet extractor until the effluent was colorless, and then subsequently extracted with ethyl acetate and ethanol in an accelerated solvent extractor, ASE350 (Dionex) [17]. The pure compounds were obtained from the bark hexane extract as previously described [18, 19] by Si-gel normal phase and reverse phase C18 chromatography. The powdered bark (<1 mm), 10 g, was mixed with 100 g water and refluxed for 20 min to mimic a common way of preparing herbal drugs, making a decoction. The decoction was filtered through glass wool when hot and lyophilized in aliquots of 1 ml, corresponding to ~10 mg dry weight.

### Quantification of dihydronitidine

The dihydronitidine content in hexane, ethyl acetate (EtOAc) and ethanol (EtOH) extracts from bark, leaf and seeds, as well as boiling water extract of the bark, was quantified by C18-HPLC. The dried extracts were dissolved by sonication (10 min) in acetonitrile (final concentration 1–10 mg/ml). The solutions were filtered (PTFT membrane, 0.22 µm) and the filtrate, 40 μl, was applied on a Chromolith Performance RP18e 100 × 4.6 mm HPLC column (Merck) attached to LaChrom Elite HPLC system (VWR-Hitachi) equipped with an L-2455 diode array detector. Elution was performed using a gradient of mobile phase A (20 mM ammonium acetate) and mobile phase B (acetonitrile) with the following time schedule: 20 % B, 0–1 min; 20–90 % B, 1–15 min; 90 % B, 15–16 min. The flow rate was 3.0 ml/min. The absorbance was recorded at 278 and 312 nm, and separation took place at 25 °C.

### Purity of dihydronitidine

The purity of dihydronitidine was estimated to be better than 97 % from ^1^H NMR spectroscopy (Additional file 1). Signals from nitidine were not detectable, and it was therefore assumed that dihydronitidine used in the in vitro assays was nitidine free. Dihydronitidine isolated from the hexane bark extract [18] was used in all assays.

### Stability of dihydronitidine in in vitro culture media

Dihydronitidine (5 µl, 10 mg/ml in DMSO) was added to 0.5 ml of RPMI 1640 complete medium supplemented with sodium bicarbonate (0.18 %) and FBS (0.5 %)(Gibco) and incubated in triplicates at 37 °C for 0, 24, 48 and 72 h (final concentration of dihydronitidine 100 µg/ml). The solution was taken to dryness in a SpeedVac vacuum concentrator (Thermo Scientific) and subsequently dissolved in 1 ml acetonitrile and sonicated for 10 min. The filtrate, 40 μl, was analysed by HPLC as described above (Quantification of dihydronitidine). Nitidine chloride (Sigma) was used as standard.

### Anti-parasitic activity in *Plasmodium falciparum*

The chloroquine susceptible strain 3D7 and the chloroquine resistant strain Dd2 parasites were cultured according to existing protocols [20]. In vitro assays are based on the method of Smilkstein et al. [21] as modified by Goodman et al. [22]. In assays longer than 72 h, the medium was changed after 72 h of parasite growth. For tests of dihydronitidine stability, drug was diluted in culture media as per standard assay protocol but incubated at 37 °C for 24 h prior to initiation of in vitro assay by addition of parasites and blood. Fluorescence was measured using an Enspire fluorescent plate reader (Perkin Elmer).

### Transmission blocking assay (ookinete conversion assay)

To assay inhibition of the early mosquito stages of parasite development, *P. berghei* gametocytes expressing green fluorescence protein (GFP) under the control of the ookinete specific promoter CTRP were induced to undergo in vitro fertilization and subsequent development to ookinete stage in a standard 96-well plate assay, essentially as described [23]. Successful development to ookinete stage was assayed by GFP fluorescence measured using an Enspire fluorescent plate reader (Perkin Elmer).

### Statistical analysis

Relative fluorescence was corrected for background and normalized to untreated controls to generate values in percent parasitaemia. Drug concentrations were converted to a log scale prior to analysis. Each in vitro assay was done in triplicate and IC_50_ values calculated using the four component model available in the Prism 6 statistical package (GraphPad). Mean and standard error values were calculated from three independent assays unless otherwise stated.

## Results and discussion

### Activity against *Plasmodium falciparum* in vitro cultures

**Fig. 1:**
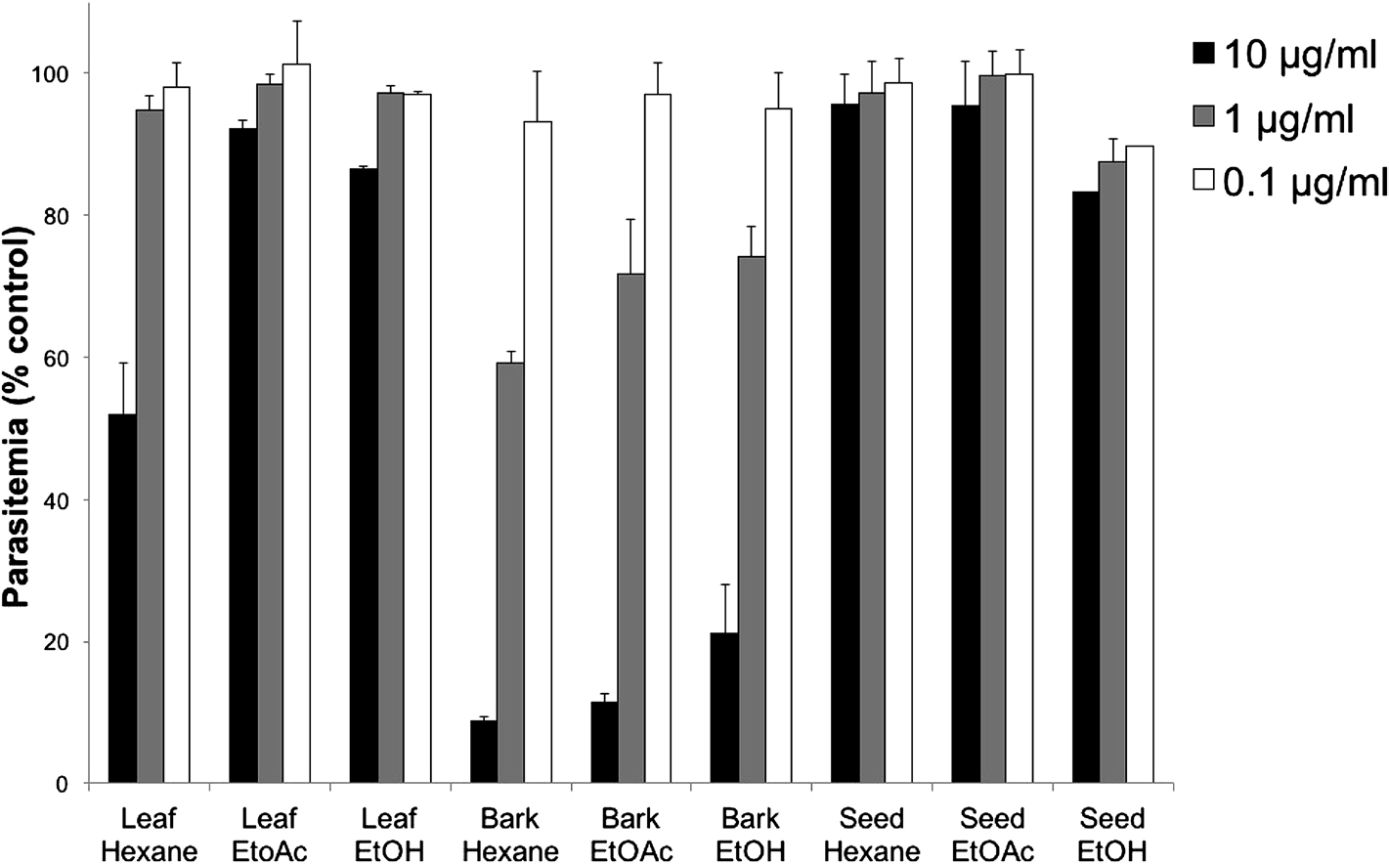
In vitro activity against *P. falciparum* of crude extracts isolated from leaf, bark and seed of *Z. heitzii*. Mean of three technical replicates ± standard deviation. *EtOAc* ethyl acetate; *EtOH* ethanol

**Fig. 2:**
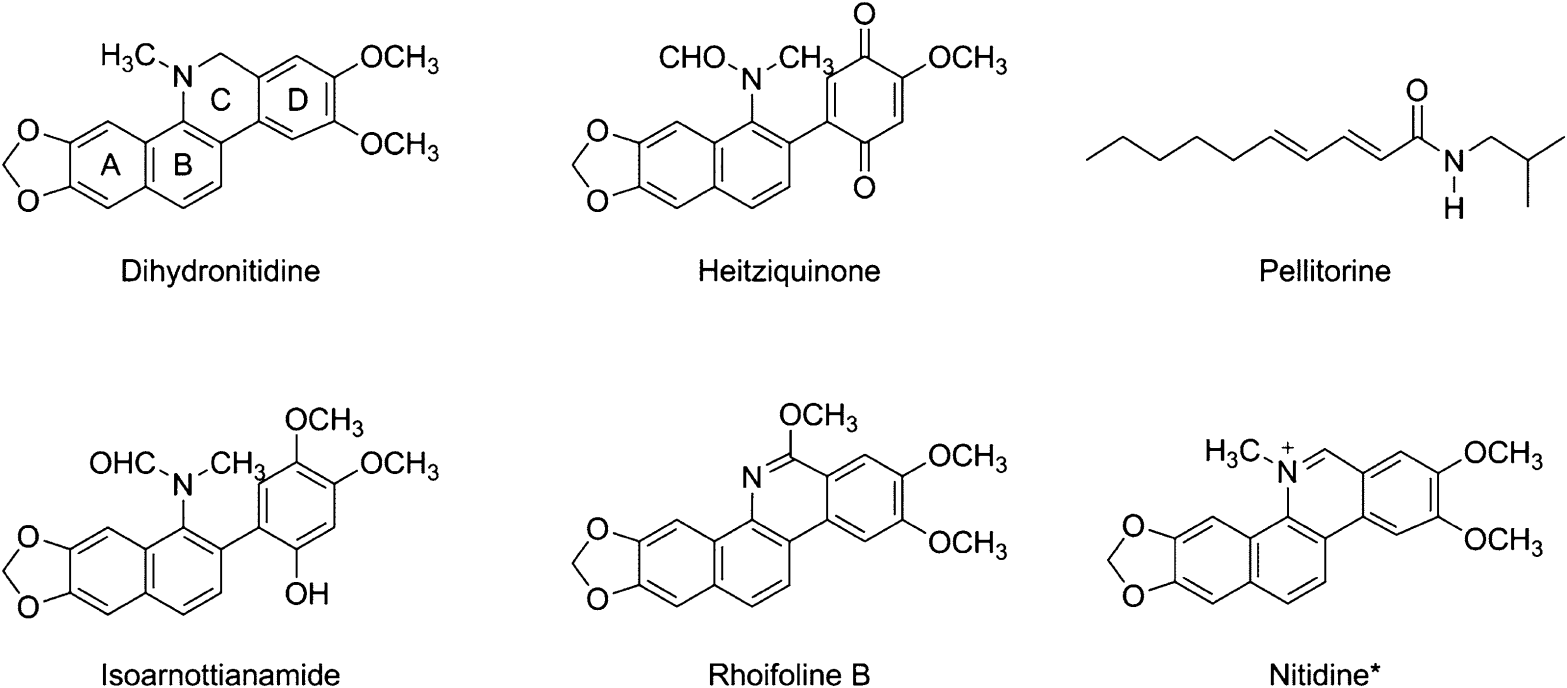
Benzophenanthridine alkaloids and pellitorine isolated from *Z. heitzii* [18, 19]. *Nitidine is included for structure comparison and was not present in the hexane extract

Extracts from the bark, leaf and seeds were screened for activity against *P. falciparum* in 48 h in vitro drug assays. The greatest activity was seen in bark extracts, regardless of the solvent, with hexane extracting the most active substances (Fig. 1). Leaf and seed extracts showed little to no anti-parasitic activity, so further analysis focused on the hexane extract of the bark. The bark extract was fractionated and the fractions tested for activity against *P. falciparum* (Additional file 2). The major components of the active fractions were purified and identified (Fig. 2) and further tested for anti-parasitic activity. The purified compounds dihydronitidine, heitziquinone and pellitorine (Fig. 2) showed significant anti-parasitic activity (Table 1), with dihydronitidine being the most active compound (IC_50_ of 0.0089 ± 0.0008 µg/ml).

**Table 1:** Inhibitory activity of hexane bark extract of *Z. heitzii* and pure compounds isolated from the extract against *P. falciparum* in 72 h assays

	IC_50_	IC_50_
	(μg/ml)	(µM)
Hexane bark extract	0.050 ± 0.004	N/A
Dihydronitidine	0.0089 ± 0.0008	0.025 ± 0.002
Pellitorine	1.96 ± 0.12	8.8 ± 0.5
Heitziquinone	3.55 ± 0.62	9.7 ± 1.6
Sesamin	>10	>28
Isobauerenol	>10	>23
Caryophyllene oxide	>10	>49
Rhoifoline B	>10	>28
Isoarnottianamide	>10	>26
Nitidine^a^	0.038 ± 0.002	0.099 ± 0.050
WR 99210^a^	3.167 × 10^−6^	1.2 × 10^−5^

Results ± SEM from three biological replicates using 3D7 parasites are shown

^a^Positive control

**Fig. 3:**
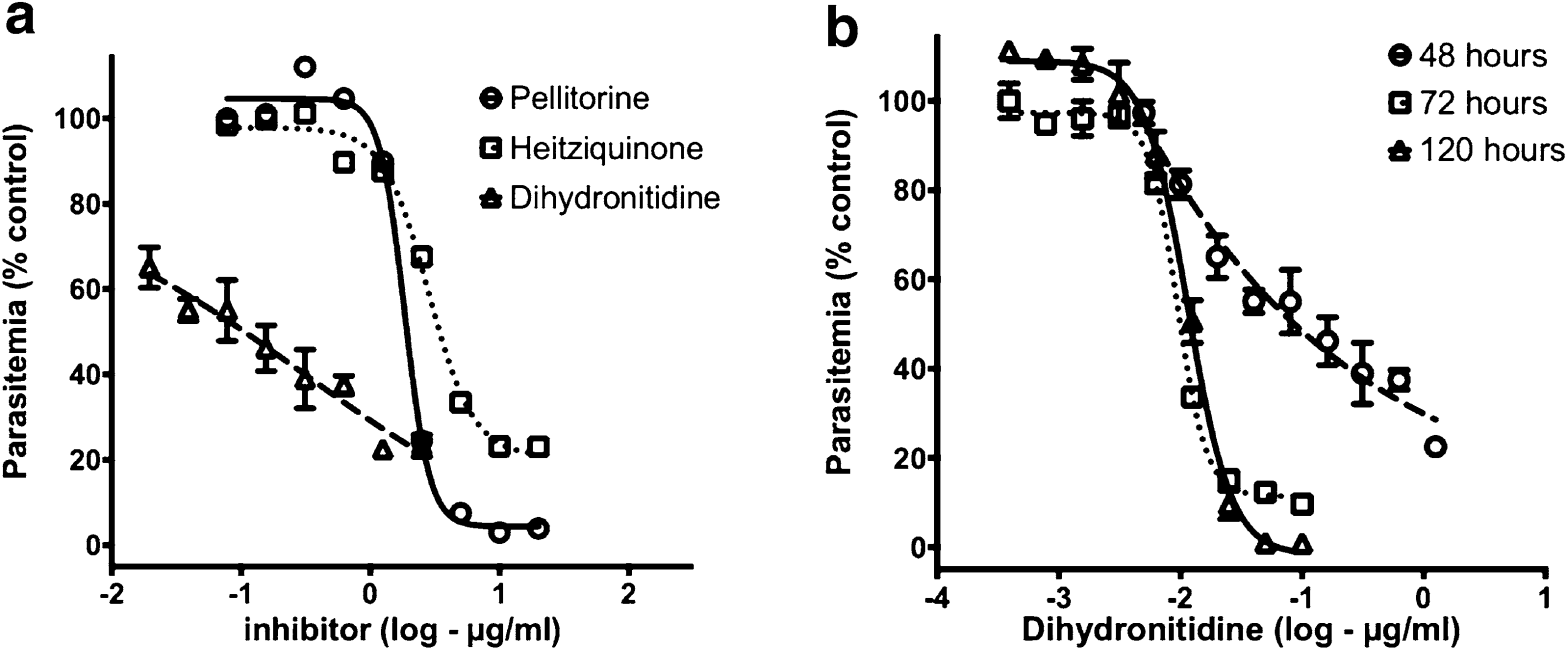
The slow acting characteristics of dihydronitidine. **a** Dose–response curves for *P. falciparum* (3D7) in 48 h in vitro drug assays showing unusual response to dihydronitidine. **b** Response of parasites to increasing time of dihydronitidine exposure. Maximum efficacy is reached at 72 h, with no improvement with longer exposures

Standard in vitro assays measuring anti-parasitic effects after 48 h exposure to dihydronitidine showed IC_50_ values of 0.3 µg/ml, which are similar to published IC_50_ values for dihydronitidine [13, 24], but with an unusually flat dose response (Fig. 3a) suggesting a slow acting drug effect. To investigate this, assays were extended with drug exposure time to 72 and 120 h. With 72 h of exposure, the IC_50_ was significantly lower (Table 1) and the drug response curves showed a typical dose response (Fig. 3b). Further increasing the length of drug exposure did not impact the effective concentration (Fig. 3b), precluding the possibility of “delayed death” drug response common to compounds targeting the parasite apicoplast [22]. Subsequent assays with hexane extracts of bark over 72 h revealed a significant reduction in effective concentration (compare Fig. 1 and Table 1), reflecting the fact that dihydronitidine comprises a significant proportion of the anti-parasitic activity of the extract.

Two other compounds extracted from *Z. heitzii* showed activity against *P. falciparum*; pellitorine and the recently reported alkaloid heitziquinone [19] (Table 1). These compounds were less active, with IC_50_ values of 1.96 ± 0.12 and 3.55 ± 0.62 µg/ml respectively, but were fast acting, having normal dose response curves with 48 h of drug exposure (Fig. 3a) and no change in effective concentration with extended drug exposure. The observed IC_50_ for pellitorine (1.96 µg/ml) is similar to previously reported values [25].

Two of the three active compounds are benzophenanthridine alkaloids, and previous studies show that the anti-malarial activity of this class of compounds is very sensitive to even small changes in chemical structure [26]. Heitziquinone has an opened C-ring and is, therefore, to the knowledge of the authors, the first open C-ring benzophenanthridine alkaloid that shows anti-plasmodial activity. This suggests that a closed C-ring is not essential for activity and that the structure–activity relationship in benzophenanthridine alkaloids is complex. A greater range of benzophenanthridine alkaloid compounds will need to be tested to elucidate these relationships.

### Stability of dihydronitidine

**Fig. 4:**
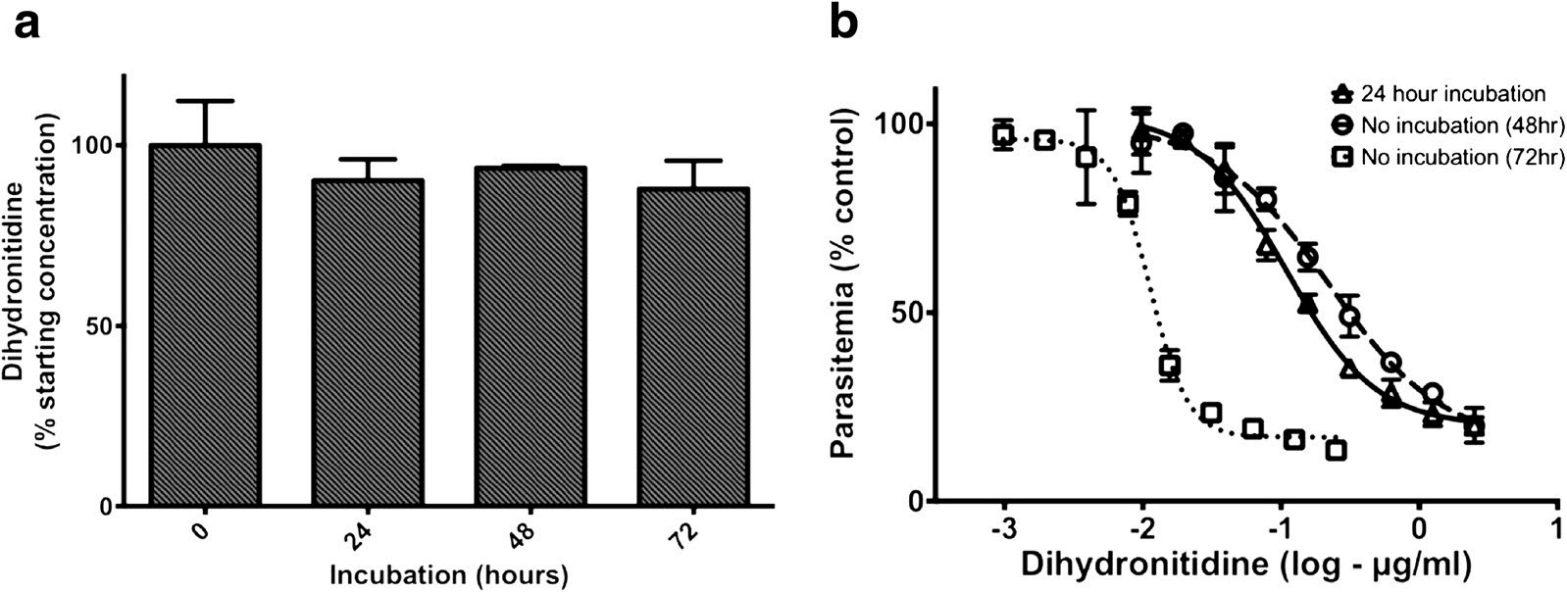
Dihydronitidine is stable under culture conditions. **a** Concentration of dihydronitidine after incubation in in vitro culture media at 37 °C showing minimal breakdown over 72 h. **b** Incubation of dihydronitidine in culture media for 24 h does not improve efficacy (mean/SD of three technical replicates)

The simplest explanation for the slow acting nature of dihydronitidine is the progressive breakdown of this compound under culture conditions to products that have greater anti-parasitic activity. Nitidine, a possible product of dihydronitidine oxidation, is thought to be the main active ingredient in several traditional anti-malarial remedies used in other parts of the world [13, 27]. The reported IC_50_ values for nitidine in standard assays (48 h) vary considerably [13, 26–28] but the lowest values fall in a similar range to those found for dihydronitidine in the long-exposure assays (72 h). It was reported previously that dihydronitidine may be slowly oxidized to nitidine under aerobic conditions [28]. To test its stability under culture conditions, dihydronitidine was incubated in parasite growth medium for 72 h at 37 °C and analysed at 24 h intervals to identify any breakdown products. Even prolonged incubation over 72 h did not significantly reduce the concentration of dihydronitidine in the sample (Fig. 4a) nor result in the production of detectable levels of nitidine. To further test whether dihydronitidine, and not its breakdown products, are responsible for the observed anti-parasitic activity, dihydronitidine was diluted in culture media in a 96 well plate according to the in vitro assay protocol and incubated at 37 °C for 24 h. Parasites were then added to initiate the in vitro assay and anti-parasitic activity was tested after 48 h. Pre-incubation had little effect on the slow acting nature of dihydronitidine (Fig. 4b) suggesting that dihydronitidine, and not its breakdown products, is responsible for the observed anti-parasitic effects.

The structural similarity between nitidine and dihydronitidine suggests that these compounds may share a mechanism of action. In this case, we would expect nitidine to show slow acting response similar to dihydronitidine. However, the observed activity of nitidine over 72 h (Table 1) was consistent with previously reported effective concentrations reported for shorter drug exposures [13, 27]. This agrees with the chemical characteristics of the compounds. While nitidine was suggested to act by binding to DNA [24], it was later reported that nitidine forms a 1-1 complex with haem in vitro and also inhibits β-haematin formation with the same potency as chloroquine [27], so it has been postulated that nitidine activity is similar to chloroquine; interfering with haemozoin formation in the food vacuole. Haem binding activity requires a positive charge, and with its quaternary nitrogen, nitidine will always be charged. Dihydronitidine, however, will not be charged at the pH of the food vacuole (pH 4.5–4.9 [29]) due to its low calculated Pkb of 2.8. This suggests that the slow-acting characteristic of dihydronitidine reflects a different mechanism of action between the two compounds. Encouragingly, neither nitidine [26] nor dihydronitidine show chloroquine cross-resistance, with the resistance index (Dd2/3D7) of 1.7 ± 0.06 for dihydronitidine and 1.26 ± 0.20 for nitidine being significantly lower than the 10.0 ± 0.17 observed for chloroquine.

### Effects on mosquito stages of parasite development

To examine transmission blocking activity, *Z. heitzii* bark hexane extract, dihydronitidine, heitziquinone and pellitorine were tested on mosquito stages of the malaria parasite life cycle for their ability to block ookinete formation in *P. berghei* (ANKA strain). As in blood stages, dihydronitidine showed the greatest activity, although it was far less potent against this stage of the parasite life cycle (Table 2). Heitziquinone had similar levels of activity against both stages but pellitorine did not inhibit ookinete development in vitro. This suggests that both dihydronitidine and heitziquinone target parasite pathways that are active early in the insect stages of the parasite life cycle and have transmission blocking activity.

**Table 2:** Inhibitory activity of *Z. heitzii* hexane bark extract, dihydronitidine, heitziquinone and pellitorine against *P. berghei* ANKA ookinete formation

	IC_50_	IC_50_
	(μg/ml)	(µM)
Hexane bark extract	1.75 ± 0.42	N/A
Dihydronitidine	0.59 ± 0.10	1.7 ± 0.29
Heitziquinone	6.20 ± 1.76	17.0 ± 4.8
Pellitorine	>20	>20

Results are the mean of three biological replicates ± SEM

### Traditional use and water extractability of active compounds

Available ethnobotanical surveys [10] indicate that bark is the only plant part from *Z. heitzii*, locally known as olon in the Republic of Congo, used for treating malaria. The presented results clearly show that the bark is more active compared to leaves and seeds, hence the rationale behind using the bark as medicine is in accordance with in vitro activity. Dihydronitidine being the main active constituent in *Z. heitzii* is supported by the low anti-parasitic potency of the seed and leaf extracts, which do not contain dihydronitidine (Additional file 3).

Traditional herbal medicines are often prepared as boiled water extracts (decoctions) and this is a common way of preparing extracts of many *Zanthoxylum* spp. used as anti-malarials [11, 15]. There is, however, little literature available describing preparations of *Z. heitzii* bark for use as an anti-malarial beyond a single report describing the dermal application of bark scrapings [10]. To explore the efficacy of *Z. heitzii* bark extracted as a traditional decoction, the levels of active compounds extracted and the in vitro anti-parasitic activity of the decoction were examined.

The dihydronitidine content in the hexane bark extract was 25.9 ± 2.4 % (HPLC), corresponding to ca. 0.6 % of the dried bark. The decoction, however, extracted low levels (~2 %) of the bark content of dihydronitidine; yielding a final concentration in the decoction of 10.7 ± 0.8 µg/ml. Other components that were extracted into boiling water were pellitorine and sesamin (Additional file 4). Pellitorine showed a relatively low anti-malarial activity, (IC_50_ 1.96 µg/ml), and sesamin was inactive (Table 1). Sesamin, however, has previously been shown to enhance activity of other compounds due to inhibition of CYP3A enzymes involved in compound metabolism [30] and this may increase the anti-malarial potency of dihydronitidine in vivo.

The activity of the lyophilized decoction against *P. falciparum* in vitro was tested. The low dihydronitidine content in a decoction of *Z. heitzii* bark was manifest in the low anti-parasitic activity. When tested against *P. falciparum* (3D7) in vitro, the decoction was only weakly effective, with an IC_50_ of 9.6 ± 1.5 µg/ml. While far less effective than the purified compounds, this does suggest that the decoction may provide a minimal amount of anti-parasitic activity due to the small amount of dihydronitidine, pellitorine and heitziquinone, and possible synergistic activity among them.

It is not entirely surprising that there is little dihydronitidine in the water decoction of *Z. heitzii* as dihydronitidine is very hydrophobic, with a calculated log S (octanol–water partitioning constant) of −4.63 at pH 7 (ACD/Labs). This makes it difficult to extract dihydronitidine using boiling water. Often in traditional medicine, a decoction is acidified using lemon juice. This is may be done to provide a more palatable taste but it also protonates the basic nitrogen often found in many alkaloids, improving their solubility in water. Dihydronitidine, however, has a calculated pKb of 2.8 ± 0.9 (ACD/Labs), suggesting it would be largely refractory to this treatment. This suggests that the decoction method is not ideal for preparing *Z. heitzii* as an anti-malarial, in contrast to a number of *Zanthoxylum* spp. containing similar, but water-soluble quaternary benzophenanthridine alkaloids.

## Conclusion

*Zanthoxylum heitzii* bark contains three compounds, dihydronitidine, pellitorine, and heitziquinone, with significant activity against *P. falciparum* in in vitro assays. Two of these compounds, dihydronitidine and heitziquinone, also show transmission-blocking activity against *P. berghei*. The most effective compound, dihydronitidine, is active in the low nM range and may be considered a lead molecule for drug development. The relatively poor extractability of dihydronitidine limits its concentrations in water preparations of *Z. heitzii*, suggesting that using preparations with non-polar extraction methods involving oil or fat, or simply using the powdered bark, could improve the anti-malarial characteristics of this traditional medicine.

## Additional files

10.1186/s12936-016-1533-x ^1^ H NMR spectrum of dihydronitidine isolated from *Z. heitzii*.
10.1186/s12936-016-1533-x Screening of fractions of *Z. heitzii* bark extract.
10.1186/s12936-016-1533-x HPLC chromatogram of *Z. heitzii* bark, leaf and seed hexane extracts.
10.1186/s12936-016-1533-x HPLC chromatogram of *Z. heitzii* water extract.
