# Erratum to: Natural products from *Zanthoxylum heitzii* with potent activity against the malaria parasite

**DOI:** 10.1186/s12936-016-1608-8

**Published:** 2016-11-17

**Authors:** Christopher Dean Goodman, Ingvild Austarheim, Vanessa Mollard, Bertin Mikolo, Karl Egil Malterud, Geoffrey I. McFadden, Helle Wangensteen

**Affiliations:** 1 School of BioSciences, University of Melbourne, Parkville, VIC 3010 Australia; 2 Department of Pharmaceutical Chemistry, School of Pharmacy, University of Oslo, P. O. Box 1068, Blindern, 0316 Oslo, Norway; 3 National Polytechnic High School, Marien Ngouabi University, BP 69, Brazzaville, Republic of Congo

## Erratum to: Malar J (2016) 15:481 DOI 10.1186/s12936-016-1533-x

After publication of the original article [1], it came to the authors’ attention that an error in the abstract was introduced during production of the article. In the Results section of the abstract, ‘pellitorine’ appears incorrectly as ‘pellitories’. The original article has been updated to correct the misspelling.
