# Supplementary material for: Natural products from Zanthoxylum heitzii with potent activity against the malaria parasite
Source: Malar J. 2016 Sep 20;15:481. doi: 10.1186/s12936-016-1533-x (PMC5029023; doi:10.1186/s12936-016-1533-x)
Supplement: Supplementary file 1 — 10.1186/s12936-016-1533-x 1 H NMR spectrum of dihydronitidine isolated from Z. heitzii. [file 12936_2016_1533_MOESM1_ESM.docx]

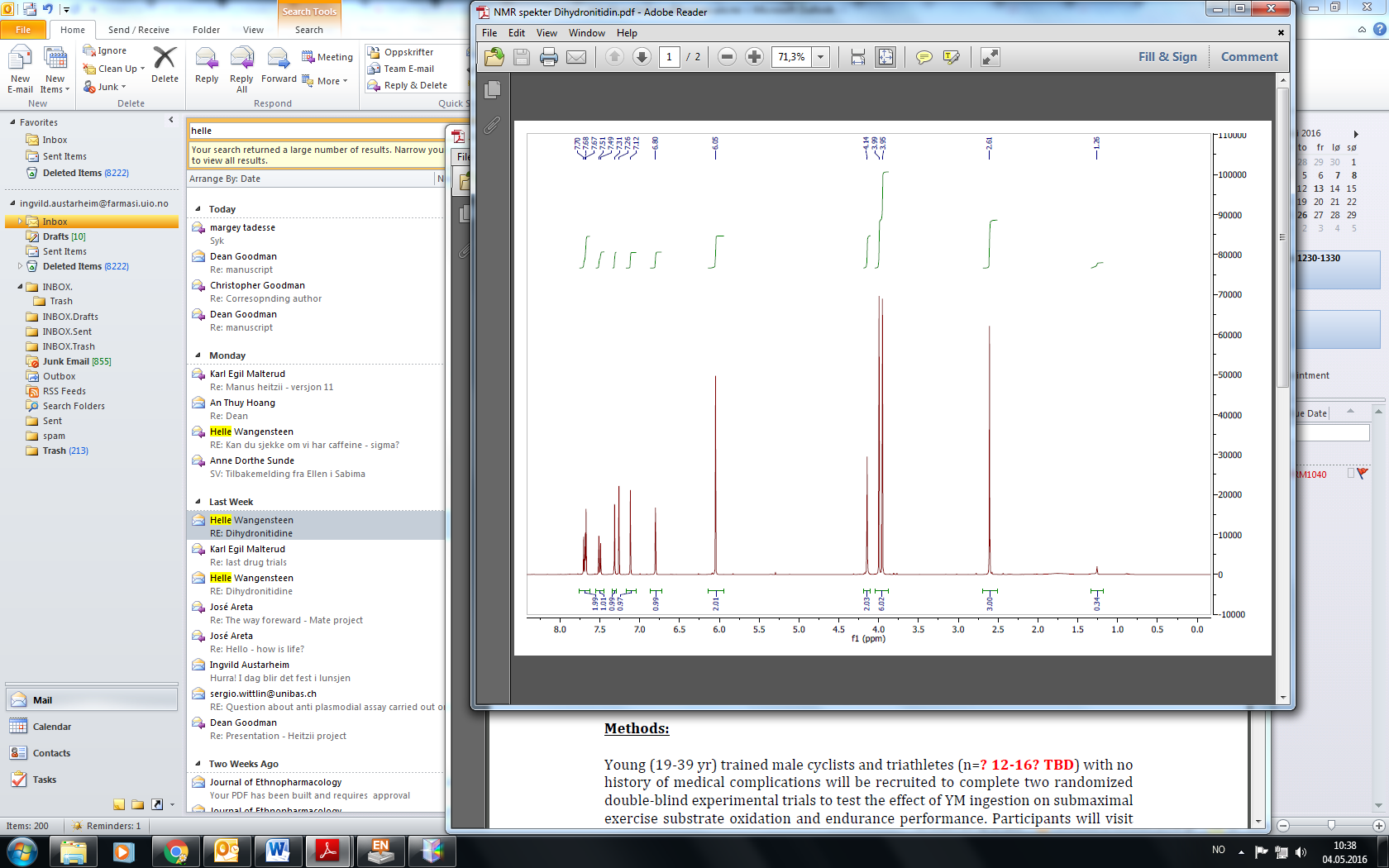


**Additional file 1**. ^1^H NMR spectrum of dihydronitidine isolated from *Z. heitzii* (CDCl_3_ was used as solvent).
