# Supplementary material for: Natural products from Zanthoxylum heitzii with potent activity against the malaria parasite
Source: Malar J. 2016 Sep 20;15:481. doi: 10.1186/s12936-016-1533-x (PMC5029023; doi:10.1186/s12936-016-1533-x)
Supplement: Supplementary file 2 — 10.1186/s12936-016-1533-x Screening of fractions of Z. heitzii bark extract. [file 12936_2016_1533_MOESM2_ESM.docx]

**Additional file 2**. Screening of fractions from *Z. heitzii* bark extract. Mean of three technical replicates.
