# Supplementary material for: Natural products from Zanthoxylum heitzii with potent activity against the malaria parasite
Source: Malar J. 2016 Sep 20;15:481. doi: 10.1186/s12936-016-1533-x (PMC5029023; doi:10.1186/s12936-016-1533-x)
Supplement: Supplementary file 3 — 10.1186/s12936-016-1533-x HPLC chromatogram of Z. heitzii bark, leaf and seed hexane extracts. [file 12936_2016_1533_MOESM3_ESM.docx]

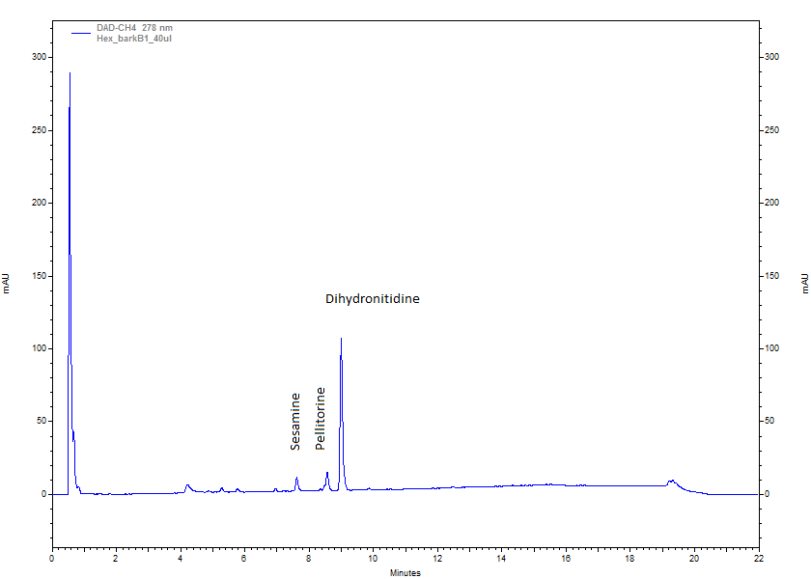


**A**


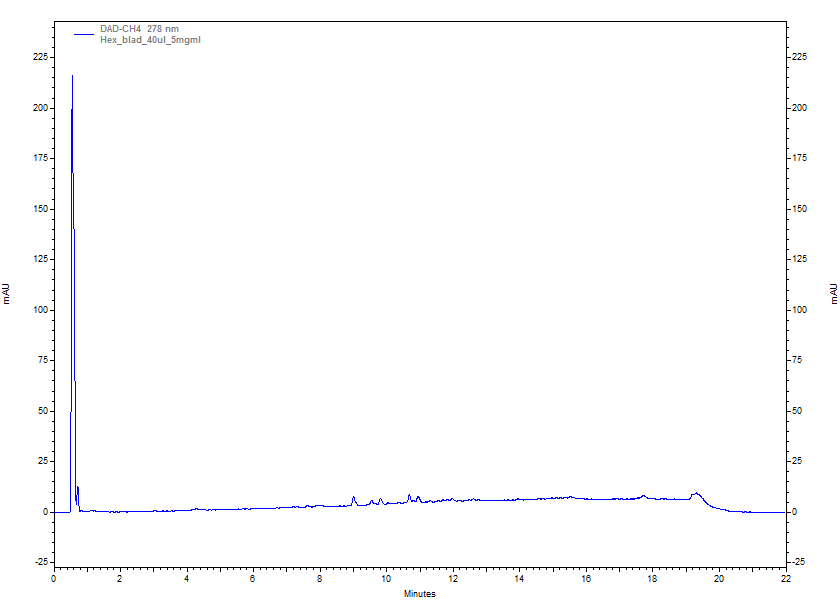


**B**


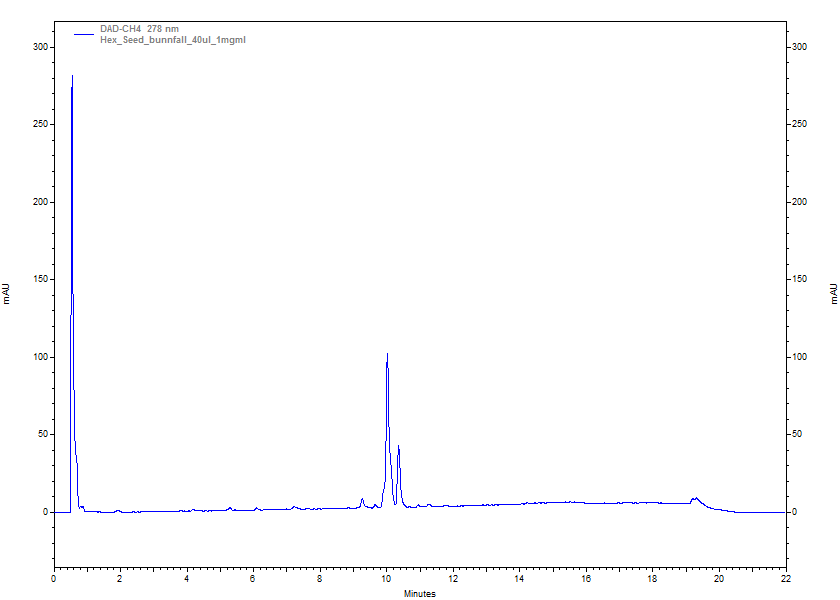


**C**

**Additional file 3**. HPLC chromatogram (278 nm) of *Z. heitzii* hexane extract of A) bark (0.05 mg/ml, 40 µl injected), B) leaves (5 mg/ml, 40 µl injected) and C) seeds (1 mg/ml, 40 µl injected).
