# Supplementary material for: Natural products from Zanthoxylum heitzii with potent activity against the malaria parasite
Source: Malar J. 2016 Sep 20;15:481. doi: 10.1186/s12936-016-1533-x (PMC5029023; doi:10.1186/s12936-016-1533-x)
Supplement: Supplementary file 4 — 10.1186/s12936-016-1533-x HPLC chromatogram of Z. heitzii water extract. [file 12936_2016_1533_MOESM4_ESM.docx]

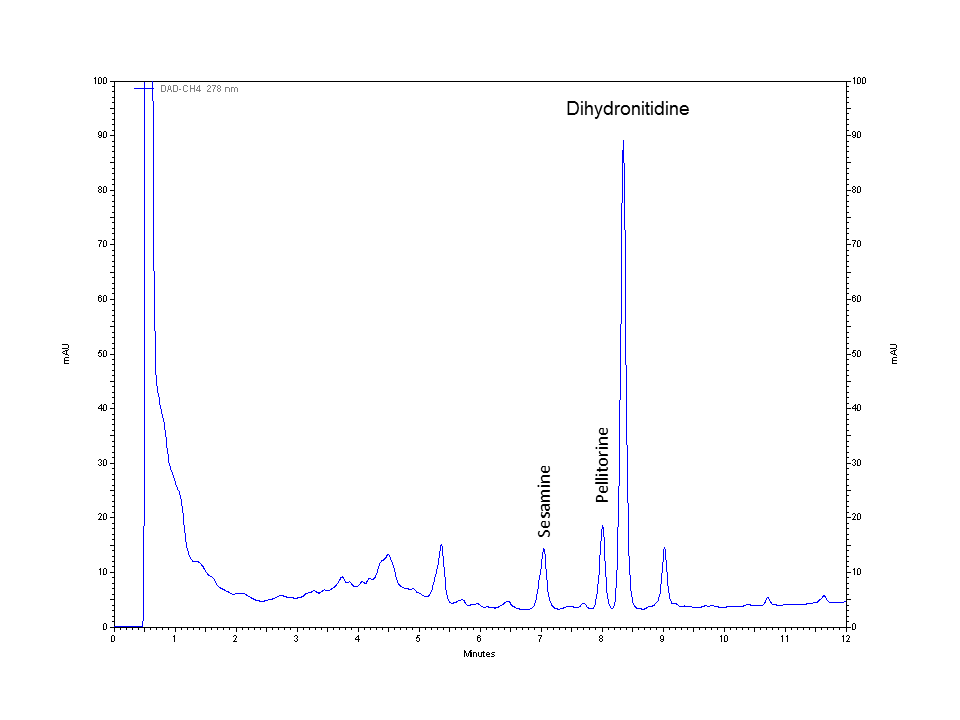
 **Additional file 4**. HPLC chromatogram (278 nm) of *Z. heitzii* water extract (decoction). The peaks from sesamine, pellitorine and dihydronitidine were based on matching retention time with authentic standards and UV spectrum (λ: 200-400nm).
